# Specific β-Turns Precede PPII_L_ Structures Binding to Allele-Specific HLA-DRβ1^*^ PBRs in Fully-Protective Malaria Vaccine Components

**DOI:** 10.3389/fchem.2018.00106

**Published:** 2018-04-06

**Authors:** Adriana Bermudez, Martha P. Alba, Magnolia Vanegas, Manuel A. Patarroyo, Manuel E. Patarroyo

**Affiliations:** ^1^3D Structure Department, Fundación Instituto de Inmunología de Colombia, Bogotá, Colombia; ^2^School of Medicine and Health Sciences Faculty, Universidad del Rosario, Bogotá, Colombia; ^3^Medicine Faculty, Universidad de Ciencias Aplicadas y Ambientales, Bogotá, Colombia; ^4^Medicine Faculty, Universidad Nacional de Colombia, Bogotá, Colombia

**Keywords:** malaria-vaccine, β-turns, structure-function, IMPIPS, LLPI, PPII_L_

## Abstract

The 3D structural analysis of 62 peptides derived from highly pathogenic *Plasmodium falciparum* malaria parasite proteins involved in host cell invasion led to finding a striking association between particular β-turn types located in the N-terminal peripheral flanking residue region (preceding the polyproline II left-handed structures fitting into the HLA-DRβ^*^ allele family) and modified **i**mmune protection-inducing protein structure induced long-lasting protective immunity. This is the first time association between two different secondary structures associated with a specific immunological function has been described: full, long-lasting protective immunity.

## Introduction

In the search for a logical and rational methodology for vaccine development, we have proposed that in-depth, chemical, physical, structural and even mathematical approaches associated with an understanding of molecules' biological functions. We have thus consistently adopted such approach working with our prototype model, the threatening and scourging *Plasmodium falciparum* malaria parasite which afflicts around 200 million people, killing nearly 600,000 of them annually, mainly children below 5 years of age in sub-Saharan Africa (World Malaria Report, 2016^1^).

This approach has involved molecular biology as well as functional analysis of the *P. falciparum* malaria parasite, enabling new molecules and their functions to be recognized (Wåhlin et al., 1984), as well as that of natural (human) and experimental (*Aotus* monkeys) host immune system molecules (Suárez et al., 2006, 2017; Lopez et al., 2014). This has facilitated the recognition of chemical, physical and structural rules regarding their interactions for tackling and resolving functional issues (Patarroyo et al., 2011). Here we show that vaccine component structural features are determinant regarding fully-protective, long-lasting protective immunity.

Vaccine components' 3D-structure can be assessed by ^1^H-NMR or X-ray diffraction; the protein structure work by many groups throughout the world has led to advances regarding knowledge about protein and peptide 3D structure and knowledge of the immune system molecules involved in major histocompatibility complex (http://www.cbs.dtu.dk/services/NetMHCIIpan/) Class I and Class II (HLA-DRβ^*^) antigen presentation to the T-cell receptor (TCR) to form the MHCII-peptide-TCR complex or immunological synapse to induce an appropriate immune response (Hennecke and Wiley, 2002; Rudolph et al., 2006). We have previously shown that **im**mune **p**rotection-**i**nducing **p**rotein **s**tructures (IMPIPS) (Patarroyo et al., 2015a) have a polyproline type II, left-handed-like (PPII_L_-like) structure to enable fitting into the appropriate HLA-DRβ1^*^ peptide binding region (PBR) (Patarroyo et al., 2012a, 2015a).

This manuscript is aimed at showing that, in the search for **l**ong-**l**asting, **p**rotective **i**mmunity (LLPI), these PPII_L_-like structures must be preceded by specific and particular β-turn structures which, when preceding other structures like α_R_-helixes and/ or other β-turn types, could also activate immune system molecules whilst inducing **s**hort-lived **p**rotective **i**mmunity (SPI) structures. When associated with PPII_L_ amino acid sequences binding to the HLA-DRβ1^*^ PBR having appropriate TCR contacting residue orientation, modified high activity binding peptides (mHABPs) can induce LLPI, which we have named IMPIPS (Patarroyo et al., 2015a; Alba et al., 2016). Specific preference for some of these β-turns and the complete absence of some others is also shown, as is the preferred association between some residues in both β-turns and PPII_L_ PBR sequences leading to high antibody titres and LLPI induction.

## Materials and methods

### Ethics statement, animal capture and study area

The current work was approved by the Fundación Instituto de Inmunología's ethics committee (FIDIC ethics committee). The capture, study, and scientific research of *Aotus* primates were authorized by the official Colombian environmental authority, CORPOAMAZONIA, (resolutions 0066/Sep/2006, 0028/May/2010, 0632/Jun/2010, 0042/Jan/2011 and 1209/Sep/2017). All animal-handling procedures were carried out according to the Guide for the Care and Use of Laboratory Animals, USA (National Research Council, 2011); such recommendations comply with Colombian regulations for biomedical research (resolution 8430/1993 and law 84/1989). CORPOAMAZONIA made a weekly visit to evaluate housing conditions, feeding regimens and the environmental enrichment of the monkeys captured. The monkeys were supervised by veterinarians and biologists; all individuals were released back into the Amazon jungle after the experimental procedures in optimal health conditions as determined by the Amazonian ethical committee and in the presence of CORPOAMAZONIA representatives.

### Synthetic peptides

Peptides were selected from several *P. falciparum* proteins as being relevant to the present study; cHABPs and mHABPs were thereby obtained by chemical synthesis. The solid-phase peptide synthesis (SPPS) method was used, following t-Boc strategy (Houghten, 1985). Cys-Gly residues were added to HABP C- and N-terminals during synthesis to enable polymerization and their complete characterization. Their purity was assessed by reverse-phase high-performance liquid chromatography (RP-HPLC) and their molecular mass was determined by mass spectrometry (MS-MALDI-TOF). Most have been synthesized in different studies throughout the last few years.

### NMR spectroscopy and structural models

This involved a protocol which has been used for several years for obtaining peptides' 3D structure by ^1^H-NMR. Native peptides (cHABPs) and their modified forms (mHABPs) from around 14 relevant proteins were prepared for ^1^H-NMR studies by dissolving ~10 mg monomer acetylated peptide in 600 μL TFE-d3/H2O 30:70% v/v. Spin systems were assigned by Double-Quantum-Filtered-Correlation SpectroscopY (DFQ-COSY) (Rance et al., 1983) and Total Correlation SpectroscopY (TOCSY) (Bax and Davis, 1985) experiments and Nuclear Overhauser Effect SpectroscopY (NOESY) (Jeener et al., 1979) ^1^H-^1^H 2D experiments. Standard spectrum procedure was used for sequential assignment. All NMR spectra were run on a Bruker DRX-600 spectrometer and processed on a computer equipped with TOPSPIN 1.3 software. All experiments were done at 295 K, except for temperature coefficients for predicting hydrogen bonds (−Δ∂HN/ΔT < 5) which were determined by TOCSY experiments, using 285, 295, 305, and 315 K temperatures.

**Figure 1:**
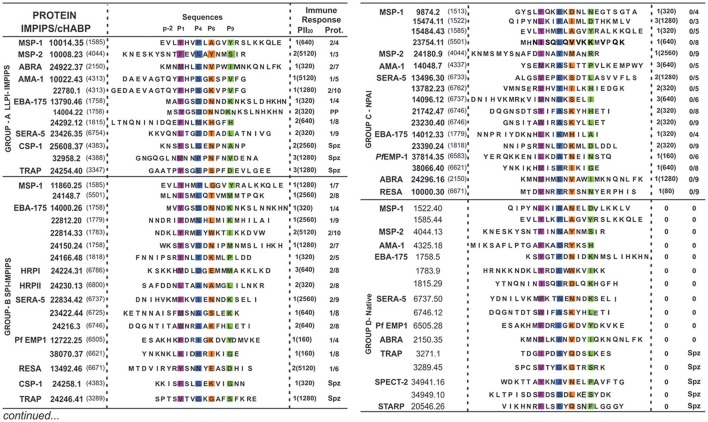
Modified and native cHABPs' chemical and immunological characteristics. *P. falciparum* merozoite (Mrz) or sporozoite (Spz)-derived proteins (in bold letters) where cHABPs (number in parenthesis) were identified, with their mHABP (in bold numbers with their associated conformed number after the dot) amino-acid sequences, highlighting PBR residues fitting into Pocket 1 (P1) in fuchsia, Pocket 4 (P4) in dark blue, Pocket 6 (P6) in orange and Pocket 9 (P9) in green, as well as the N-terminal PFR (–p2) preceding PBR residues essential in β-turn formation. Regarding immune response, the amount of monkeys producing immunofluorescence antibody (IFA) titres is shown in parenthesis, along with the amount of fully-protected monkeys (absolute absence of parasites in their blood during challenge). PP: partial protection. Group A (LLPI-IMPIPS), group B (SPI-IMPIPS), group C (NPAI) and group D (native) peptides. Spz peptide-induced protection could not be tested since there are no reliable *Aotus* monkey-adapted *P. falciparum* strains.

Distance constraints were extracted from NOESY spectra for obtaining structural models at 295 K temperature. All NOE intensities were converted into distance ranges as strong (1.8–2.8 Å), medium (2.8–3.5 Å) or weak (3.5–5.0 Å), along with a.rstrnt file. These, together with .inp, .car, .mdf input files and Insight II package (Accelrys, Inc, Software, USA), or .upl, .lol, .cya and .aco input files and Cyana software were used for calculating peptide structures. Distance geometry (DGII) software was used for building a set of conformers for 50 structures; these structures were then refined by using a simulated annealing protocol (Discover and Cyana software). Structures having low energy and NOE violations greater than 0.30 Å and no angle violations greater than ≥2.8° were then selected. The final distinctive low energy family, was assembled from consensus conformer lowest energy and superimposing the structured regions' backbones on structures obtained by ^1^H-NMR restrictions. The structure chosen have been indicated by our serial numbers and is shown after the dot (Figure 1).

### Classifying β-turns

**Figure 2:**
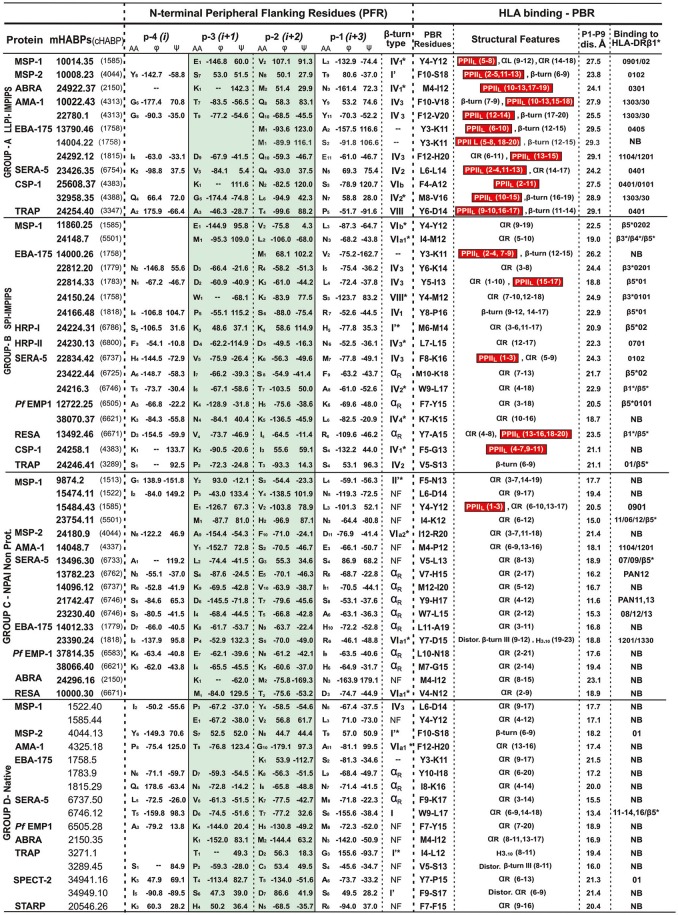
β-turn types and αR structures in the N-terminal PFR region preceding the PPII_L_ or other structures in PBR sequences. Previously classified by immunological results, A, B, C and D group peptides having residues located in the N-terminal PFR region (their amino acid and position shown in subscript in the central column) ϕ and ψ angles (in degrees °) classified according to (de Brevern, 2016) green highlighting relevant positions –p3 and –p2 in β-turn formation, αR or random structures (NF, not found). The next column describes the β-turn or NF structures, preceding residues fitting into PBR P1 to P9 of the HLA-DR molecules they bind to (last column). Red highlights (in parenthesis) residues in the structural features column forming PPII_L_ regions, followed by distance in Å between fittings into PBR P1 to P9. NB in the last column = not binding to any HLA-DR molecule, according to the netMHCIIpan 3.1 platform. Please note that all LLPI-IMPIPS (group A) PPII_L_ structures were preceded by specific β-turn types.

The N-terminal peripheral flanking region (PFR) adjacent to the PBR has been analyzed for many of the ~300 peptides studied by ^1^H-NMR; however, only 62 cHABPs and mHABPs have been included in this study due to the other peptides having been classified into different groups (Figure 2), having similarity regarding both their immunological and structural behavior. Peptides representative of each group were thus selected and are shown here. Chimera and Insight II software were used for structural analysis, measuring ψ and ϕ dihedral angles (Pettersen et al., 2004) and the distances between Cα_*i*_ and Cα_*i*+3_ residues for each peptide (Reyes et al., 2017a). Different β-turns have been classified in our peptides according to recent work on dihedral angles (de Brevern, 2016), where ϕ_i+1_, ψ_i+1_, ϕ_i+2_ and ψ_i+2_ β-turns were taken into account: −60.00, −30.00, −90.00, 0.00 for type I turn, 60.00, 30.00, 90.00, 0.00 for type I', −60.00, 120.00, 80.00, 0.00 for type II, 60.00, −120.00, −80.00, 0.00 for type II', −120.0 130.0 55.0 41.0 for type IV_1_, −85.0 −15.0 −125.0 55.0 for type IV_2_, −71.0 −30.0 −72.0 −47.0 for type IV_3_, −97.0 −2.0 −117.0 −11.0 for type IV_4_, −60.00, 120.00, −90.00, 0.00 for VI_a1_, −120.00, −120.00, −60.00, 0.00 for VI_a2_ −135.00, 135.00, −75.00, 160.00 for VI_b_ and −60.00, −30.00, −120.00, 120.00 for type VIII. It has been accepted that 3 angles are allowed to have ±30° deviation for β-type grouping and the fourth ±45°. β-turns which were found to be further apart were classified as distorted (marked by an asterisk ^*^).

### HLA-DRβ1^*^ and IMPIPS

The NetMHCIIpan 3.1 algorithm (for predicting peptide binding to MHC-II molecules) was used for predicting HLADRβ^*^ binding characteristics. This was based on the quantitative MHC class II binding capability of more than 100,000 peptides analyzed. Peptides binding to specific HLA-DRβ^*^ alleles with high affinity (~95% specificity and 90% sensitivity) were accurately predicted (90%), as were correct HLA-DR peptide binding cores (previously determined by X-ray crystallography). This algorithm recognized peptides having very high theoretical binding to specific HLA-DRβ1^*^ alleles and alternative β-chain isotypes, like HLA-DRβ3^*^, β4^*^ and β5^*^ alleles (Andreatta et al., 2015).

CLIP-binding HLA-DRβ1^*^0301 [PDB code: 1A6A, (Ghosh et al., 1995)] and HA-binding (hemagglutinin peptide) HLA-DRβ1^*^0401 [PDB code: 1J8H, (Hennecke and Wiley, 2002)] located in these molecules' PBR were used in this paper as templates for superimposing **32958**.35 onto CLIP and **25608**.37 onto HA. This was aimed at ascertaining the formation of H-bonds stabilizing the p-MHCII complex and highlighting interaction between α-chain-HLA-DRβ1^*^ and the –p2 residue in the N-peripheral flanking region (β-turn fragment). The UCSF Chimera program was used for obtaining these measurements.

### Immunization and challenge

Immune response data and HLA-DR genotypes acquired from immunization studies of *Aotus* in groups of 5 to 8 spleen-intact monkeys for each peptide and each group have been previously described (Patarroyo et al., 2015a). Briefly, the *Aotus* monkeys were immunized with 250 μg polymerized peptide with Freund's complete adjuvant for the first dose and Freund's incomplete adjuvant for the second and third doses. Blood was used for immunological analysis on day 1 before (PI) the first immunization and 20 days after the first (I_20_), second (II_20_) and third (III_20_) immunizations.

LLPI-IMPIPS was assessed by re-challenging *Aotus* monkeys having high antibody titres and positive protection (defined as the complete absence of the parasite in a monkey's blood stream) 60 days after the end of the first trial in which a monkey proved fully-protected (Bermúdez et al., 2014; Alba et al., 2016).

## Results and discussion

Jardesky et al., using X-ray crystallography 20 years ago (Jardetzky et al., 1996), demonstrated that HLA-DRβ1^*^-associated endogenous peptides had a PPII_L_ structure (found later on in both antigenic and immunogenic peptides); these structures fit perfectly well into MHCII PBR (Dessen et al., 1997; Fremont et al., 1998).

Our group found that a specific group of immune protection-inducing protein structure (IMPIPS, involving LLPI and SPI) had or contained PPII_L_-like structures in our search for a logical and rational methodology for malaria vaccine development (Patarroyo et al., 2012a,b, 2015a). Modified HABPs (mHABPs) had been developed against highly-virulent *P. falciparum Aotus*-adapted FVO strain lethal intravenous challenge; they were derived from **c**onserved **h**igh **a**ctivity **b**inding **p**eptides (cHABPs) from proteins directly involved in the parasite's invasion of a host (hepatocytes, endothelial or red blood) cells (Patarroyo et al., 2017). This was the first demonstration that chemically-synthesized, vaccine-induced immune protection was associated with a particular 3D structure; (Patarroyo et al., 2015a). As the ideal one should specifically bind to HLA-DRβ1^*^ alleles, it was called Group A, inducing LLPI and having 26.5 ± 1.5 Å distance between residues 1 to 9 fitting into HLA-DRβ1^*^ PBR (Alba et al., 2016), all having or containing PPII_L_-like structures. This confirmed that PPII_L_-like conformation is an absolute requirement for LLPI induction. Group B induced **s**hort-lived **p**rotective **i**mmunity (SPI) whose structure in the PBR binding region (HLA-DR residues 1 to 9) was ~3.5Å shorter, preferentially binding to HLA-DRβ3^*^, β4^*^ or β5^*^ haplotypes (Alba et al., 2016). Group C consisted of **n**on-**p**rotective **a**ntibody-**i**nducing (NPAI) mHABPs which were shorter in the PBR binding region and group D consisted of native cHABPs which did not induce antibody production or protection (Figure 1) or binding to any Class II molecule when used as immunogens, according to the netMHCIIpan 3.1. platform (Andreatta et al., 2015) (Figure 2).

Vey recently we demonstrated N-terminal peripheral flanking residue (PFR) (Reyes et al., 2017b) preference for amide (Q, N), sulfur-containing (M) and one having β-branched apolar (V) or large aliphatic (L) residues in IMPIPS position –p2. We have also shown preference for charged (E, K, R, D, H) and short polar (S, T) residues in SPI mHABP position –p2 (Reyes et al., 2017b).

This observation prompted us to look for an association between such immunological functions and particular 3D structures, applying Francis Cricks catch-phrase, “If you do not understand function, study structure and vice versa.” After having obtained ~300 3D structures for these peptides by powerful ^1^H-NMR spectroscopic analysis (600 MHz), we searched for an association between these characteristics and particular 3D structure conformation in our peptides (Patarroyo et al., 2011, 2012a,b, 2015a,b; Bermúdez et al., 2014; Alba et al., 2016; Reyes et al., 2017a).

Briefly, PPII_L_ are particular secondary structures in globular proteins and peptides, having left-handed geometry involving 3-5 amino acids, 9.3 Å pitch distance and three to five residues per turn, all amide bonds ideally having *trans* conformation (ω = 180°), an absence of internal H-bonds, side-chains almost perpendicular to a peptide's backbone and ϕ ~75° ± 25 and ψ ~145° ± 25° dihedral angles (Creamer, 1998; Stapley and Creamer, 1999; Adzhubei et al., 2013; Zondlo, 2013). Many of them contain prolines (~70%) (Chellgren and Creamer, 2004); however, ~40% of them do not (Kumar and Bansal, 2016) and a hexaproline peptide 3D structure confirmed the aforementioned physicochemical characteristics (Wilhelm et al., 2014).

β-turns must have a < 7.5Å distance between Cα_*i*_ and Cα_*i*+3_ for residues involved in the turn (Hutchinson and Thornton, 1994), central residues are not helical and their ϕ and ψ angles define the β-turn type where, according to Brevern, a deviation of ± 30° from the canonical values is permitted for 3 of the 4 angles and ± 45° for a fourth one (Fuchs and Alix, 2005; de Brevern, 2016). Originally, Venkatachalam (1968) defined types I, II and III with their corresponding mirror images I', II' and III'; later on, Lewis added V and V' (Koch and Klebe, 2009) and Hutchinson et al., (Hutchinson and Thornton, 1994) divided VI into VI_a1_, VI_a2_ and VI_b_ and precisely defined type VIII. Type VI is characterized by a *cis*-Pro in position *i*+*2* and type VII is associated with a kink. The frequently occurring (~35% of all β-turns) and highly undefined type IV group was recently subdivided into types IV_1_, IV_2_, IV_3_, IV_4_, and IV miscellaneous (IV_misc_) (de Brevern, 2016), observing some structural superimposition with previously-determined β-turn types (Madan et al., 2014).

Some turns, like type III' (1.5%), V (0.03%) and V' (0.02%), have recently been discarded due to their low frequency or their structural similarity with 3_10_ helixes (e.g., type III and type I' similar to type III') (de Brevern, 2016).

We thus classified the PBR N-terminus peripheral flanking region groups as IMPIPS (LLPI, SPI), NPAI mHABPs and native cHABPs. It was striking that 23/29 (79%) SPIs or LLPI-IMPIPS exhibiting protection against challenge (Figure 1), had β-turn structures preceding HLA-DRβ1^*^, β3^*^, β4^*^ or β5^*^ Class II binding residues in their PBRs (Figure 2). It was observed that group B (SPI-IMPIPS) was much more diverse, having β-turn types IV_1_, IV_2_, IV_3_, IV_4_, VI_a1_, VI_b_, VIII (de Brevern, 2016) and three (3) αR structures, while group A was more selective regarding β-turn preference, having β-turns IV_1_ (similar to I'), IV_2_, IV_3_, VI_b_, VIII (Figure 2). Two EBA-175 (1758) cHABP-derived LLPI-IMPIPS in group A (**13790**.46 and **14004**.22) were called short, since a perfect β-turn structure could not be assigned as only two amino acids preceded the PBR; this IMPIPS had M in position –p2 (Figure 2). It was striking that all IMPIPS (Group A) had β-turns structures. The preceding β-turn was very short for SPI-IMPIPS having PPII_L_ regions in one of them (**14000**.26); another was preceded by an α_R_ region and another had the PBR in the αR region (Figure 2). It was clearly observed that most (7/17) NPAI (group C) had αR, type II'^*^, ${\text{VI}}_{\text{a}1}^{*}$ or ${\text{VI}}_{\text{a}2}^{*}$ β-structures (5/17), whilst another large group (6/17) had random structures (i.e. β-turn not found – NF) (Figure 2).

The situation in group D (native) was quite similar, as most native cHABPs had type I or I' (4/16), αR (3/16), VI_a1_ and IV_3_ β-turn (one each); interestingly, (6/16) had random structures (NF) in the preceding region, though sequence PBR binding by ^1^H-NMR had α_R_ structures (theoretically, the netMHCpan 3.1 platform predicted that only 3/16 had some HLA-DR binding) (Figure 2). Such data clearly correlated 3D structural conformation with particular immunological outcomes where IMPIPS inducing LLPI had or contained PPII_L_-like structures in the region fitting into the PBR preceded by specific β-turn types.

Interestingly β-turns I', IV, IV_b_, and VIII preceded PPII_L_ structures (Figure 2, highlighted in red) in most IMPIPS. This data showed LLPI-IMPIPS clear preference for type IV_1_ to IV_3_ and VIII β-turns while the most frequently occurring β-turns I (38.21%), II (11.81%), II' (2.51%), and IV_misc_ (~17%) accounted for ~70% of β-turns (Koch and Klebe, 2009; de Brevern, 2016) not being present, suggesting that β-turns I, II, II', and IV_misc_ are not associated with immune protection.

This was exceptional information since it showed the association of 2 different types of secondary structures to provide a defined functional role: LLPI induction (Figure 2, group A). We would like to stress that LLPI definition has been based on re-challenging *Aotus* monkeys having absolute protection (the complete absence of parasites in their blood) after immunization with these IMPIPs, the 1st and 2nd challenges mimicking what occurs in hyper-endemic areas where inhabitants may suffer as many as 18 infectious bites per night or repeated malaria episodes.

As previously stated (Reyes et al., 2017b), we have been increasing the amount of molecules analyzed. Group A (LLPI-IMPIPS) had a clear preference in –p2 for amide residues Q (4/12) and N (2/12), sulfur-containing M (3/12) and short alcohol (T), aliphatic amino acids (L) or β-branched apolar (V) amino acids, one per group. Group B (SPI-IMPIPS) had a clear preference in position−2 for charged residues, like K (5/17), R (1/17), H (1/17) and D (1/17), short alcohol S and T (2/17 each), β-branched I (2/17) and V (1/17) with contributions from L and M (1/17 each), whilst no single SPI-IMPIPS had amide (Q, N) residues in –p2. Group C preference in –p2 was clearly for T and S (3/17 each), with contributions from N and V (2/17 each), polar residues like Y, H, K, E and apolar residues like F, M, G (1/17 each) while p10 had a slight preference for E (4/17) and I (3/17) with contributions from M and R (2/18 each) and T, L, Y, V, A, and S (1/17 each). Group D native cHABP had no preference for residues in –p2 (Figure 2).

These results add quite interesting structure-function association-related information, since it has been suggested that IV_3_ and IV_4_ are very different regarding dihedral angle distribution but having similar amino acids composition. We found that this characteristic held true for position –p3 where D was present in most (4/7) IV_3_ in both IMPIPS groups; residues containing amides like Q and N were predominant in –p2 for LLPI-IMPIPS (6/12) (Figure 2, Group A), while positively-charged residues (K, H and R) were predominant in SPI-IMPIPS (7/17) (Figure 2, Group B). Proline was under-represented regarding β-turns in our mHABPs, since it has been suggested that type VI β-turns have cis-proline in position –p2 (we did not find this in our few type VI peptides). Type IV_1_ was closer to β I' than a β II turn; IV_3_ and IV_4_ were closer to β I turn, having very similar amino acid but very different structural conformation (de Brevern, 2016). It is striking that Q was only present in 4/12 group A (LLPI-IMPIPS) mHABPs in –p2 (Figure 2), while absent in the other 50 mHABPs from other groups, suggesting a very relevant role for these residues in LLPI-IMPIPS.

Despite similarities regarding dihedral angle distribution, our IMPIPS had very different amino acid composition to that described by de Brevern (2016), suggesting a different, distinctive amino acid composition but similar secondary structure conformation for peptides involved in protective immune responses.

**Figure 3:**
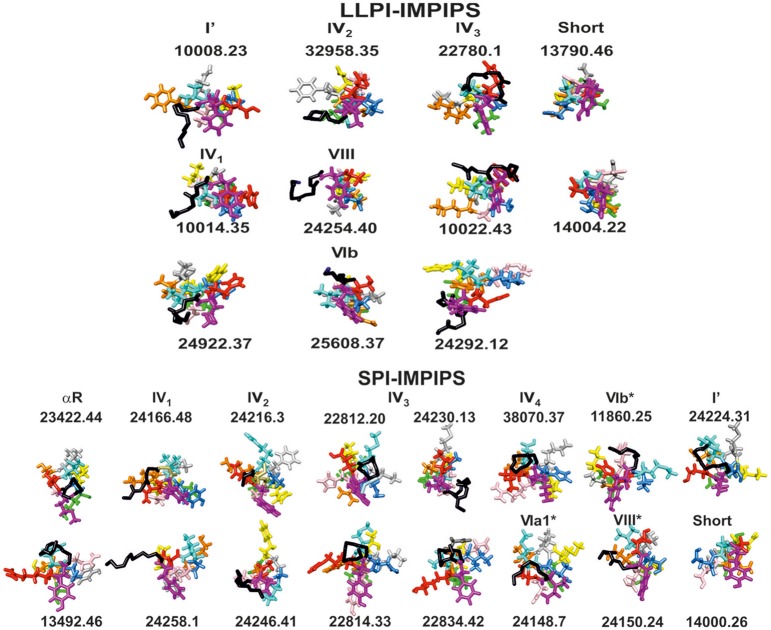
Front view of groups A (LLPI-IMPIPS) and B (SPI-IMPIPS); conformers determined by ^1^H-NMR. Color codes are the same as those for residues fitting into P1 (fuchsia), position p2, p3 (cyan), P4 (dark blue), p5 (pink), P6 (orange), p7 (gray), p8 (yellow), P9 (green). Residue positions (p) are those contacting the TCR. Black sticks highlights the backbone of residues forming β-turn types or αR structures preceding amino acid fitting into the PBR. Note that most N-terminal structures were left-hand orientated suggesting interaction with the HLA-DR α-chain to collaborate in stabilizing this complex. Note also the total absence of the most frequent β-turn types like I, II, II' and IV_ori_, accounting for ~70% of β-turn types.

Figure 3 shows the front view for β-turns in IMPIPS (LLPI and SPI) 3D structure (displayed in black sticks), most pointing toward the left-hand side, suggesting that once mHABPs have been anchored to HLA-DRβ1^*^, β3^*^, β4^*^ or β5^*^ PBR pocket 1 (P1), these specific β-turn sequences could interact with other specific residues located in Class II molecule α-chain. Group A LLPI-IMPIPS also had p2 pointing upwards and toward the right-hand side, while p3(cyan) also pointed upwards and toward the left-hand side (displaying a *gauche*+ orientation); exactly the opposite occurred in SPI-IMPIPS, confirming the critical role of these TCR-contacting residues' orientation in LLPI induction (Bermúdez et al., 2014; Alba et al., 2016).

**Figure 4:**
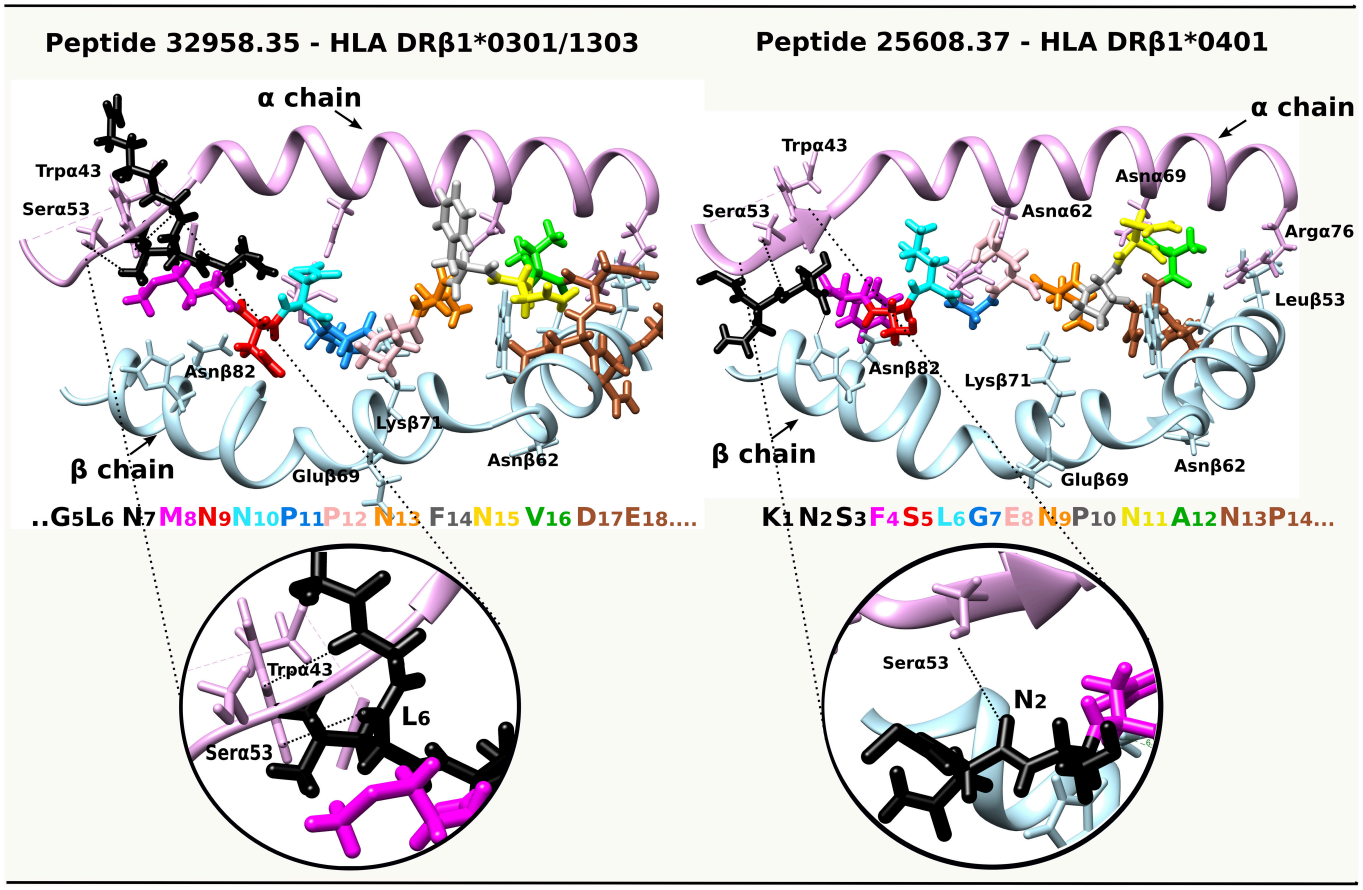
IMPIPS **32958**.35 interaction with HLA-DRβ1^*^0301 and IMPIPS **25608**.37 interaction with HLA-DRβ1^*^0401. DRβ1^*^' α-chain contact with N-terminal peripheral flanking residues (–p2 in black) are shown by a dotted line. The colors of residues in each IMPIPS are taken from the code for Figure 3 and MHC II α (pink ribbon) and β-chain residues (blue).

A tempting hypothesis is that IMPIPS mHABPs interact via their PPII_L_ preceding β-turn structures with Pheα51, Alaα52, Pheα54 and Trpα43 residues located in the MHCII molecule 3_10_-helix (Yin and Stern, 2013) establishing H-bonds between residues in –p2 backbone atoms and Trpα43 and Serα53 side-chain and backbone atoms or interactions between the aforementioned peptide and α-chain-MHCII side-chain hydrogens. NetMHCPan 3.1 (Andreatta et al., 2015) predicted **32958**.35 IMPIPS binding to HLA-DRβ1^*^1303; however, we were able to superimpose **32958**.35 onto HLA-DRβ1^*^0301 (Ghosh et al., 1995) due to sequence and haplotype similarity; this complex has been shown to have interactions between L6:O and Hγ:Serα53 and L6:Hδ1 and HZ2:Trpα43 (Yin and Stern, 2013) and **25608**.37 has been shown to have H-bonds between N2:O and Hγ:Serα53 and **32958**.35 (Figure 4). They collaborate in stabilizing peptide binding to the PBR, specifically when fitting into P1 where peptides having low binding affinity and kinetic instability are highly susceptible to HLA-DM-mediated peptide exchange (Wieczorek et al., 2017). The opposite has been clearly demonstrated, as epitope selection is constrained by favoring the presentation of peptides having longer HLA-DM-mediated half-lives (Yin et al., 2012).

Two more facts should be highlighted. All these LLPI-IMPIPS (Group A), with one (**10008**.23, having 23.8 Å) exception, had 24.1Å to 29.1 Å distance (26.5 Å ± 2.5 Å) between residues fitting into P1 to P9 of the HLA-DRβ1^*^ PBR of the allele to which they specifically bound. This was different to what occurs with SPIs-IMPIPS (Group B) having a 18.7 Å to 24.4Å distance between the same residues (21.6 Å ± 2.5 Å), preferentially binding to β3^*^, β4^*^, β5^*^ allelic families (Figure 2) (Alba et al., 2016).

This distance was shorter (11.6 Å to 21.4 Å) in NPAI mHABPs (group C), some binding to HLA-DRβ1^*^ alleles but mainly proceeded by α-helix structures (7/17), II'^*^, ${\text{VI}}_{\text{a}1}^{*}$, ${\text{VI}}_{\text{a}2}^{*}$ β-turns (4/17), or not found (NF) structures (6/17). The distance was even shorter in native cHABPs (Group D), very few binding to some HLA-DR alleles (3/16) (Figure 2), thereby confirming the critical role of distance between residues fitting into P1 to P9 in the HLA-DRβ1^*^PBR.

Another striking physicochemical and immunological characteristic was found in IMPIPS mHABPs; no particular β-turn type was found to be associated with any HLA-DR allele or allele family (Figure 2).

Polar residues like Q, N or T, or large aliphatic residues like L in –p2 were associated with the highest antibody titres, while those having V or M in this position had lower antibody levels, suggesting a preference for such amino acid in this position (–p2) for stabilizing this complex and antibody production. There was a strong association between residues fitting into P1, like F and L with Q and N in position –p2, while Y in P1 was mainly associated with V, M or T in –p2.

It has been clearly shown here that type IV_1_ had more classical characteristics, being closer to type I' β-turns, that type IV_2_ and VIII were structurally very close, having high amino acid sequence similarity, that IV_2_ seemed to be a less extended form of VIII and that type IV_3_ seemed to be a half-turn, as shown here. This is why these β -turns have been grouped in Figure 3.

What we have shown here is that PPII_L_-like structures must be preceded by specific β-turn types to induce an LLPI-IMPIPS, speeding up the long-sought-after process of vaccine design for humankind's health and welfare.

## Author contributions

AB and MEP: conceived and supervised the study; AB, MV, and MPA: analyzed data; AB, MEP, and MPA: wrote the manuscript; MEP and MAP: made manuscript revisions. All authors contributed to the discussion of this manuscript.

### Conflict of interest statement

The authors declare that the research was conducted in the absence of any commercial or financial relationships that could be construed as a potential conflict of interest.

## Footnotes

^1^http://www.who.int/en/

## Abbreviations

MHC: Major histocompatibility complex
TCR: T-cell receptor
HLA: Human leukocyte antigen
IMPIPS: Immune protection-inducing protein structure
PPII_L_: Polyproline type II, left-handed
PBR: Peptide binding region
LLPI: Long-lasting, protective immunity
SPI: Short-lived protective immunity
cHABP: Conserved high activity binding peptide
mHABP: Modified high activity binding peptide
NPAI: Non-protective antibody-inducing
PFR: Peripheral flanking residue
Mrz: Merozoite
Spz: Sporozoite
IFA: Immunofluorescence assay.
